# Immunogenicity and protection effects of cationic liposome containing imiquimod adjuvant on leishmaniasis in BALB/c mice

**DOI:** 10.22038/ijbms.2019.35739.8515

**Published:** 2019-08

**Authors:** Ahmad Mehravaran, Maryam Rezaei Nasab, Hadi Mirahmadi, Iraj Sharifi, Ebrahim Alijani, Amin Reza Nikpoor, Javad Akhtari, Mansure Hojatizade

**Affiliations:** 1 Infectious Diseases and Tropical Medicine Research Center, Resistant Tuberculosis Institute, Zahedan University of Medical Sciences, Zahedan, Iran; 2 Department of Parasitology and Mycology, Faculty of Medicine, Zahedan University of Medical Sciences, Zahedan, Iran; 3 Leishmaniasis Research Center, Kerman University of Medical Sciences, Kerman, Iran; 4 Clinical Immunology Research Center, Zahedan University of Medical Sciences, Zahedan, Iran; 5 Immunogenetic and Cell Culture Department, Immunology Research Center, School of Medicine, Mashhad University of Medical Sciences, Mashhad, Iran; 6 Immunogenetics Research Center, Department of Medical Nanotechnology, Faculty of Medicine, Mazandaran University of Medical Sciences, Sari, Iran; 7 Department of Basic Medical Sciences, Neyshabur University of Medical Sciences, Neyshabur, Iran

**Keywords:** Cationic liposome Leishmania major, Imiquimod, Immune response, Vaccine

## Abstract

**Objective(s):**

Protection against leishmaniasis, in the murine model, is dependent on developing a potent CD4^+^ mediated Th1 type response. Liposomes can be applied as immunoadjuvants to stimulate immune responses to different antigens. In the present study, it was investigated whether DOTAP liposomes having SLA and imiquimod adjuvant, can induce a Th1 response and protect against *Leishmania* *major* challenge in BALB/c mice.

**Materials and Methods:**

Liposomes were provided applying the lipid film procedure. BALB/C mice were subcutaneously immunized, three times with 2-week intervals, with various formulations. Assessment of lesion development and parasite burden in the foot and spleen after challenge with *L. major*, assessment of Th1 cytokine (IFN-γ), and titration of IgG isotypes assessed the type of generated immune reaction and the protection extent.

**Results:**

The mice immunized with Liposome DOTAP+imiquimod+SLA showed smaller footpad swelling which was meaningfully different (*P<*0.05) compared with other groups. The highest level of IgG2a was observed with Lip DOTAP+imiquimod+SLA more than the control (*P<*0.001). Mice immunized with Lip DOTAP+SLA+imiquimod demonstrated the least number of live parasites in the footpad and spleen. Cytokine assay showed that the greatest IFN- γ secretion was seen in the splenocytes of mice immunized with all formulations as compared to the control group (*P<*0.0001). In contrast, the lowest IL-4 production was detectable in Lip+imiquimod+SLA spleen, which was not significantly different compared with other groups.

**Conclusion:**

The results of this study show that liposome DOTAP+SLA+imiquimod formulation generates a cellular immune response that is protective against challenge against *L. major*.

## Introduction

Leishmaniasis is a major public health problem with an increasing pattern of disease burden. It is a poverty-related ailment that influences the poor and is associated with poor housing, illiteracy, malnutrition, weakness of the immune system, displacement, gender discrimination, and lack of resources. It is estimated that 350 million people are at the risk of infection with *Leishmania *parasites, with a reported worldwide prevalence of approximately 12 million cases. Leishmaniasis has been classified as one of the most elective diseases, and the World Health Organization has considered it as a critical epidemic-prone parasitic infectious disease affecting the disadvantaged and the poor (1). 

Despite developments in molecular and pharmaceutics immunology, there is no satisfactory chemotherapy for cutaneous leishmaniasis. The existing chemotherapy for visceral leishmaniasis (VL) is generally efficient in immunocompetent patients, but it is expensive and not free from side effects known as leishmaniasis. Available drugs require multiple injections and demonstrate limited effects practically in some endemic regions; there is no licensed and efficient vaccine introduced against the disease so far (2, 3).

Few experimental *Leishmania *vaccines have been assessed in clinical trials. In general, the approaches adopted show restricted prophylactic effectiveness because of different reasons, such as lack of a proper adjuvant, an antigen, and the delivery system (4, 5). From among different generations of vaccines against leishmaniasis, only the first generation vaccines (killed parasite components) in a phase-3 clinical trial have been evaluated, with the most effective vaccines being associated with that generation. First generation vaccines containing the whole-killed parasites have been proposed to control the disease. However, most of the vaccine studies focus on the second vaccines consisting of recombinant proteins, polyproteins, or dendritic cells fraught with peptides derived from leishmanial antigens (6).

Considering the promising results obtained from leishmanization, it seems that crude *Leishmania* antigens, such as SLA (soluble *Leishmania* antigens), which are made of plenty of antigen epitopes, including gp63, KMP-11, PSA, gp46, etc, are the prospective candidates for preparing the vaccine (2, 7). In this study, SLA has been selected to generate immunity to leishmaniasis, as a model of the first generation vaccine and an immunogenic agent. 

Protection against leishmaniasis, at least in the murine model, is dependent on developing a potent CD4^+^ mediated Th1 type reaction specified by the elevated titers of IgG2a, activation of the CD8^+^ T cell population, and high level of IFN-γ generation (8, 9). In contrast, the production of a Th2 type of an immune response is associated with the exacerbation and susceptibility of the ailment. Based on the studies on the life cycle and the pathogenic mechanism of *Leishmania major*, dendritic cells (DCs) and macrophages that are the main antigen-presenting cells (APCs) of the skin affect the development of the cellular immune reaction against *Leishmania* significantly (10).

To enhance the persistence of antigens, immune stimulation, as well as the presentation, uptake, and co-administration of safe and effective adjuvants, are required. The combination of immunopotentiating adjuvants and delivery systems is a favorable approach to the rational vaccine design (11, 12). Numerous nanocarriers, such as liposomes, polymersomes, micelles, archaeosomes, and ISCOMs have so far been utilized to deliver protein antigens to professional APCs (13-20). Liposomes, i.e., the bilayer vesicle encapsulating aqueous contents, are used as the delivery systems for peptides, drugs, proteins, and DNA. Besides, liposomes may be utilized as immunoadjuvants to stimulate immune reactions in different antigens. Antigens can be associated with liposomes in several ways, including the encapsulation of the antigen within the aqueous core of the liposome, through the lipid bilayer and transmembrane regions of the antigen, and via surface adsorption (21, 22). Cationic liposomes are more stimulating and promising as delivery vehicles because of their clinical use safety, low immunogenicity, depot effects, and simplicity of preparation. DOTAP liposomes interact with negatively charged molecules on the surface of antigen-presenting cells (APCs) and target antigens for more effective phagocytosis (23-28). 

The capability of improving the immune response of vaccines through some compounds was first demonstrated with aluminum salts, titled ‘adjuvants,’ added to attenuated or killed pathogens. Adjuvants are classified into delivery systems and immunostimulants. Immunostimulants interact with special receptors, such as TLRs (Toll-like receptors) and others, while delivery systems trigger the immune reaction through multiple mechanisms, based on their specific properties (23, 24). Imiquimod adjuvants are currently used to treat warts for this molecule through interaction with TLRs. Besides, the presence of imiquimod in the vaccine formulation when administered together with the antigen can enhance the immune response. Also, imiquimod exerts its immune-modulating effects by triggering the generation of pro-inflammatory cytokines, like IL-12 and IFN- γ. The immune reaction effects of the production of cytokines associated with the Th1-induced response increase in IgG2a. (28). 

The purpose of the present research was to examine whether DOTAP liposomes with SLA and the imiquimod adjuvant fusion as the candidates for combining the vaccine would be capable of inducing a Th1 type of reaction and protection against the *L. major* challenge in BALB/c mice, or not.

## Materials and Methods

***Animals, ethics statement***

The experiment was performed on female 6–8 week old BALB/c mice in the Laboratory of Animal Research Center of Zahedan University of Medical Sciences. The mice were kept in the animal care equipment in pathogen-free conditions. The protocol of experimental design was confirmed by the Institutional Ethical Committee and Research Advisory Committee of Zahedan University of Medical Sciences (Education office dated March 31, 2010; proposal code, 88527), on the basis of the Specific National Ethical Guidelines for Biomedical Research issued by the Research and Technology Deputy of Ministry Of Health and Medicinal Education (MOHME) of Iran.

***Parasites, imiquimod, and soluble Leishmania antigen (SLA)***

*L. major* strain (MRHO/IR/75/ER) employed in the current research was applied previously in leishmanization and to prepare an experimental *Leishmania* vaccine and the leishmanin test in Iran (25, 26). Imiquimod (R837) was provided by Invivogen Company. The SLA preparation was done using the established protocol with some alterations. In brief, the parasites were harvested at stationary phase and rinsed three times using the HEPES buffer (10 mM+sucrose 10%, pH 7.4) (27). Afterward, the number of promastigotes was set to 1.2 × 10^9^ per ml in a buffer having an enzyme inhibitor cocktail, 50 μl/ml (Sigma, St Louis, MO, USA). The parasites were then lysed by the freeze-thaw procedure accompanied by probe sonication in an ice bath. The supernatant of the centrifuged lysate parasites was gathered, dialyzed against HS buffer solution, and sterilized by passage through a 0.22 μm membrane and kept at −70 ^°^C. The SLA protein concentration was indicated by BCA (bicinchoninic acid) protein assay kit (Thermo Scientific, USA)(29). 

***Liposome preparation and characterization***

Liposomes were provided by the lipid film procedure. The lipid phase having 1, 2 dioleoyl propyl 3 trimethylammonium bromide (DOTAP) (20 mM; Avanti polar lipids, USA) and cholesterol (10 mM; Avanti polar lipids, USA) (2:1 molar ratio) were dissolved in chloroform in a sterile tube. The solvent was removed using rotary evaporation (Hettich, Germany), causing deposition of a thin lipid film over the tube’s wall. The lipid film was then freeze-dried (TAITEC, Japan) overnight to remove the solvent. The lipid film was hydrated and dispersed in a sterile buffer (HEPES buffer 10 mM pH 7.4) having SLA (2 mg/ml). The multilamellar vesicles (MLVs) were converted to unilamellar vesicles under argon employing a bath sonicator (Bandelin, Germany) at 45 ^°^C for 15 min. The dispersion of liposome was extruded 13 times via 400 nm polycarbonate membranes (Avestin, Canada). The zeta potential and particle size of liposome preparations were measured by the Dynamic Light Scattering Instrument (Nano-ZS, Malvern, UK). Particle sizes were indicated as the mean±standard deviation and polydispersity index (PDI) (n=3). Zeta potentials were reported as the means±zeta deviation (n=3) (21). 

***Characterization of the prepared formulations***

The SLA concentration encapsulated in liposomes was indicated by the BCA protein assay kit (Thermo Scientific). Analytical SDS-PAGE was done to qualitatively calculate the SLA encapsulated in the liposomal SLA (Lip-SLA). The discontinuous system included running and stacking gel of 1 mm thickness (12.5% and 4.78% w/v acrylamide, respectively). The electrophoresis buffer was 25 mM Tris, 192 mM glycine, and 0.1% SDS at pH 8.3. Electrophoresis was done for 45 min at 140 V constant voltages. The same SLA amount (2.5 or 5 g) was loaded to every well for various formulations. The gels were stained with silver to detect protein after electrophoresis (30). 

***Immunization of BALB/c mice***

As stated in the introduction, the main goal of the study was looking into whether DOTAP-based liposome formulations with SLA and imiquimod adjuvant fusion could stimulate the immune system. Therefore, different vaccine formulas were chosen accordingly. Various mice groups, ten mice in each, were immunized subcutaneously (SC) three times at a 3-week interval in the footpad (RF) with one of these formulations: HEPES buffer, SLA, Lip DOTAP, Lip+ imiquimod, and Lip+ imiquimod+SLA, in a final volume of 50 µl.

***Challenge with Leishmania major promastigotes***

For this experiment, 1×10^6^ late stationary phase *L. major* promastigotes in 50 μl volume were inoculated SC into the right footpad of immunized and control mice two weeks after the last booster injection. Lesion progression was weekly accompanied by measurement of the thickness of the infected footpad in comparison with the same footpad thickness before infection employing a digital caliper (Mitutoyo Measuring Instruments, Japan) (31). 

***Quantitative parasite burden after challenge***

Viable parasites, spleens, and footpads from mice vaccinated with *L. major* were harvested in every treatment group for identification. The viable *L. major* parasites in the spleen and footpad of mice were obtained by restricting the dilution assay procedure (31, 32). The mice were killed at week six after challenge. The feet were removed aseptically and homogenized in RPMI 1640 supplemented with 2 mM glutamine, 10% v/v heat-inactivated FCS (Eurobio, Scandinavie), 100 units of penicillin per ml, and 100 μg/ml of streptomycin sulfate (RPMI-FCS). The homogenate was diluted with the media in eight serial 10-fold dilutions and put in every well of flat-bottom 96-well microtiter plates (Nunc, Denmark), having solid layer of rabbit blood agar in tetraplicate and incubated for 7–10 days at 25±1 ^°^C. The negative and positive wells (absence and presence of the motile parasite, respectively) were identified by an inverted microscope (CETI, UK). The viable parasites in every spleen and infected footpad were indicated using the GraphPad Prism software, a statistical procedure to limit the dilution assay.

***Antibody isotype assay***

The levels of antigen-specific serum IgG subclasses were indicated through a standard enzyme-linked immunosorbent assay (ELISA) technique. Samples of blood were obtained from mice before and eight weeks post challenge and the sera were separated and stored at –20 ^°^C. The evaluation of IgG1, anti-SLA IgG total, and IgG2a was carried out to identify bound antibodies (33). Microtiter plates (Nunc, Denmark) were covered with 50 μl of SLA (10 μg/ml) in PBS buffer (0.01 M, pH 7.3) and serum serial dilutions overnight at 4 ^°^C. HRP-rabbit anti-mouse IgG isotype was administered to the plates based on the manufacturer’s protocol (Invitrogen Inc, USA). Optical density (OD) was indicated at 450 nm by 630 nm as the criterion wavelength.

***ELISpot assay***

The ELISpot assessment was done using mouse ELISpot kits from U-cytech (Utrecht, the Netherlands). At week 2, three mice from every group after the last booster injection (before challenge) were killed. Their splenocytes were separated and restimulated *in vitro* via mitogen Concanavalin A (Con A) as a positive control or SLA as a recalled antigen. ELISpot plates were covered with antibodies of anti-IL-4 or anti-IFN-γ and incubated overnight at 4 ^°^C. The splenocytes (5×10^5^ cells/well) were cultured in triplicate in 200 μl volume with DMEM (as background responses), medium having Con A (as positive controls), or medium having 10 μg/ml of SLA in pre-coated plates. Spot counting was conducted using the Kodak 1D software (Version 3.5, Eastman Kodak, Rochester, New York) after incubation (37 ^°^C, 5% CO2) for 24 hr (for IFN-γ assay) or 48 hr (for IL-4 assay). The average number of spots±SD in triplicate wells was estimated and demonstrated as spot-forming units (SFU) per 105 splenocytes.

***Flow cytometry***

For identification of cellular uptake of formulations, splenocytes were separated two weeks after the last booster and stained for intracellular cytokine IFN-γ (anti-IFN-γ–FITC) and IL-4 (anti-IL-4-FITC) based on BD protocols Cytofix/Cytoperm™ and Fixation/Permeabilization Kit. Splenocytes (10^6^ cells/ml) in medium having GolgiPlug™ (1 μl/ml) were triggered with PMA/ionomycin cocktail (2 μl/ml) at 37 ^°^C for 4 hr. One hundred five splenocytes were added to flow cytometry tubes after stimulation and rinsed twice with stain buffer (2% FCS in PBS). One microliter anti-CD8a-PE-cy5 antibody and 1 μl anti CD4-PE-cy5 antibody in isolated tubes were used to stain splenocytes at 4 ^°^C for 30 min. The cells were rinsed with stain buffer and fixed using Cytofix/Cytoperm™ solution. The fixed cells were rinsed twice using Perm/Wash™ buffer and stained with 1 μl anti-IFN-γ- FITC antibody at 4 ^°^C for 30 min. CD4 cells were stained with 1 μl anti-IL-4-PE antibody. The cells were rinsed with Perm/Wash™ buffer and suspended in 300 μl stain buffer for flowcytometric analysis Calibur (BD Biosciences, USA).

***Statistical analysis***

GraphPad Prism software was used to record and analyze the data. One-way ANOVA assessed the variations among different groups. Regarding significant F-value, Tukey–Kramer multiple comparisons were done as a post-test to evaluate the average values in various mice groups. *P*<0.05 was assumed as statistically meaningful.

## Results

***The characterization of liposome***

**Figure 1:**
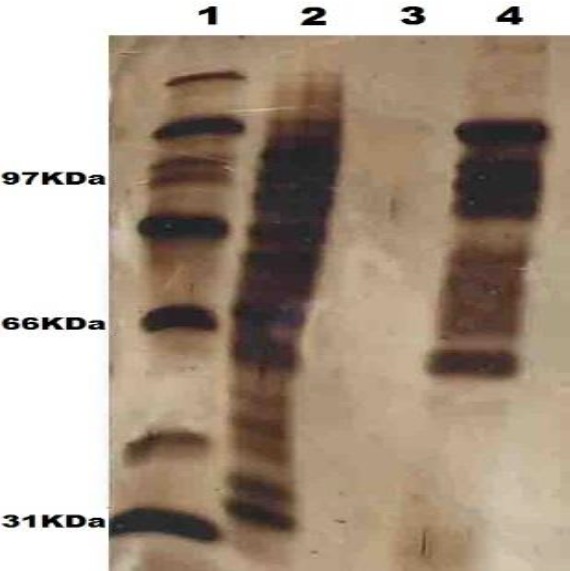
SDS-PAGE analysis; Lane 1: Low-range protein standard (Sigma, USA); Lane 2: Soluble *Leishmania* Antigen; Lane 3: Empty Liposome; Lane 4: Liposome+Imiquimod+Soluble *Leishmania *Antigen

Lip+SLA+imiquimod, Lip+imiquimod, as well as empty liposome compositions’ average diameter values were 322.5±8, 245.8±9, and 211.7±7 nm; the zeta potentials were 40.8±4 and 49.9±3 and 57.3±6 mV, respectively (n=3), indicating that all various formulations are almost homogenous. The entrapment of SLA in liposomes was estimated at 82.6±4.2% (n = 3). Before injection, the concentration value of SLA in the compositions was set at 50 μg/50 μl. Liposomal SLA and SLA were characterized using SDS-PAGE electrophoresis (Figure1). The analysis of SLA SDS-PAGE showed different protein bands with various ranges. The liposomal SLA analysis showed that nearly every band was similar to the free SLA, indicating that SLA proteins got captured in the composition following the liposome preparation.

***Challenge results***

**Figure 2:**
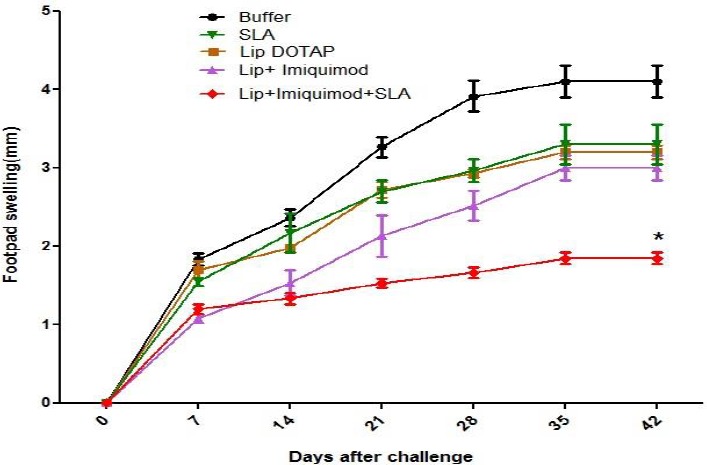
Footpad swelling in BALB/c mice immunized subcutaneously, three times in 3-week intervals, with Soluble *Leishmania* Antigen, Liposome DOTAP, Liposome+Imiquimod, Liposome+Imiquimod+ Soluble *Leishmania* Antigen or buffer alone. The footpad thickness of each mouse was measured on both footpads for 42 days. Each point represents the average increase in footpad thickness±SEM (n=7). At weeks six after challenge, the lesion sizes in mice immunized Liposome+imiquimod+Soluble *Leishmania* Antigen was significantly (*P<*0.05) smaller compared with the other groups

The development of the lesion was controlled through the estimation of footpad thickness, on a weekly basis (Figure 2). The size of the lesion developed abruptly in the mice that were SLA or buffer immunized contrary to the mice that were immunized using Lip DOTAP, Lip+ imiquimod, and Lip+ imiquimod+SLA following the challenge. In week 6 following the challenge, the lesion sizes in the mice that were immunized with Lip+ SLA + imiquimod were by far (*P*<0.05) smaller than those of other groups. 

***The post-challenge parasite burden in the footpad***

**Figure 3:**
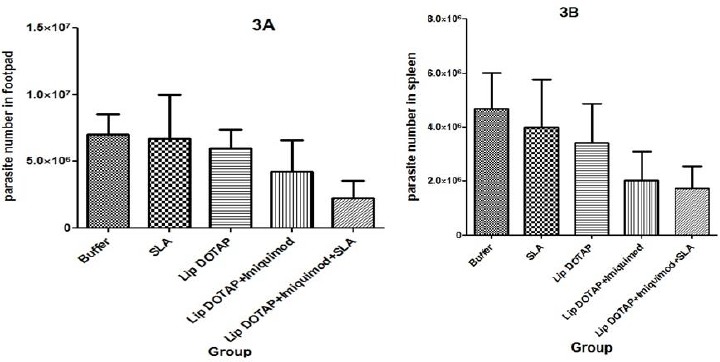
Footpad parasite burden (3A) and spleen parasite burden (3B) in BALB/c mice. Mice immunized subcutaneously, three times in 3-week intervals with Soluble Leishmania Antigen, Liposome DOTAP, Liposome+Imiquimod, Liposome+Imiquimod+Soluble *Leishmania *Antigen or buffer alone after challenge with *L. major* promastigotes. A limiting dilution analysis was performed after challenge on the cells isolated from the spleen and foot of individual mice and cultured in tetraplicate in serial 8-fold dilutions. The wells were assessed microscopically for *Leishmania major* growth, and the number of viable parasite per spleen was determined by GraphPad Prism5 software. The bar represents the average score±SEM (n=3)

The viable *L. major* numbers were estimated in the footpad of various groups of infected mice, 47 days after the challenge (Figure 3A). The mice that were immunized by SLA+imiquimod+Lip showed the lowest burden of parasites compared to others, but no meaningful variation was observed in the parasite numbers in all groups that were vaccinated, contrary to the control group (*P*>0.05).

***The spleen’s parasite burden***

The viable *L. major* parasites’ number was calculated inside the spleen of various mouse groups on day 42 following the challenge (Figure 3B). The mice immunized by SLA+imiquimod+Lip demonstrated fewer live parasites contrary to other groups of mice. However, no meaningful difference was observed in the splenic parasite numbers in vaccinated groups in comparison to the control. 

***Antibody reaction***

**Figure 4:**
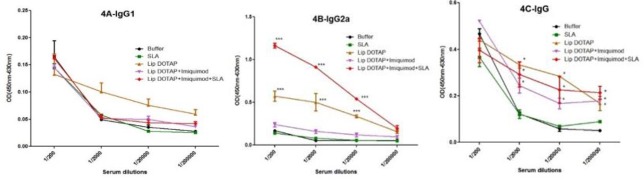
The levels of anti- Soluble *Leishmania* Antigen specific IgG1 (A), IgG2a (B), and IgG (C) antibodies based on mean absorbance in sera of BALB/c mice before challenge. Mice immunized subcutaneously, three times in 3-week intervals, with Soluble *Leishmania* Antigen, Liposome DOTAP, Liposome DOTAP+ Imiquimod, Liposome DOTAP+Imiquimod+Soluble *Leishmania* Antigen or buffer alone. Blood samples were collected from the mice 2 weeks after the last booster. The assays were performed using ELISA method in triplicate at 200, 2000, 20,000, or 200,000-fold dilution for each serum sample. Values are represented as mean ± SD. * indicates significant difference between the groups, *, *P<*0.05; ***, *P<*0.001

**Figure 5:**
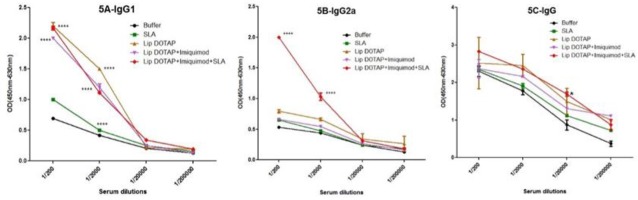
Levels of anti- Soluble *Leishmania* Antigen specific IgG1 (A), IgG2a (B), and IgG (C) in sera of BALB/c mice; Mice immunized subcutaneously, three times in 3-week intervals, with, Soluble *Leishmania* Antigen, Liposome DOTAP, Liposome DOTAP+Imiquimod, Liposome DOTAP+Imiquimod+ Soluble *Leishmania* Antigen, or buffer alone; Blood samples were collected from the mice 2 weeks after the last booster and 8 weeks after challenge. The anti-Soluble *Leishmania* Antigen IgG1, IgG2a, and IgG levels were assessed using ELISA method. The assays were performed in triplicate at 200, 2000, 20,000, or 200,000-fold dilution for each serum sample. Values are represented as mean ± SD. * indicates significant difference between the groups, *, *P<*0.05; ****P<0.0001

In order to determine the type of immune reaction produced, IgG anti-SLA antibodies specific to IgG2a, IgG1, and the subclasses of IgG got titrated prior to (Figures 4 A-C) and following (Figures 5 A-C) the mentioned challenge. Before the challenge, as Figures 5 A–C show, a meaningful (*P*<0.001) variation existed in the levels of IgG2a in Lip DOTAP as well as Lip DOTAP+imiquimod+SLA. Besides, a meaningful (*P*<0.05) difference of IgG Abs existed in the sera of the mice that were immunized by Lip DOTAP, Lip DOTAP+imiquimod, or Lip DOTAP+imiquimod+SLA in comparison to the controls receiving HEPES buffer. The maximum level of IgG2a was seen in the sera of the mice immunized by Lip SLA+imiquimod+DOTAP; the level was significantly (*P*<0.001) higher than that of the control group that received the HEPES buffer. Besides, a meaningful difference (*P*<0.05) existed among the group of mice immunized by Lip DOTAP, Lip DOTAP+ imiquimod, and Lip DOTAP+imiquimod+SLA and the group receiving HEPES buffer in terms of IgG antibody; however, no meaningful difference was observed in the level of IgG1 between the vaccinated groups compared to the control group.

There was a significant difference in the levels of IgG Abs, IgG2a, and IgG1 in the sera of the mice that were immunized by different formulations in comparison to the controls receiving the HEPES buffer after the *L. major* promastigote challenge (Figures 5A-C).

The sera of the mice immunized by Lip SLA+imiquimod+DOTAP generated considerably (*P*<0.0001) the greatest antibody titer of IgG2a compared to the groups receiving the buffer of HEPES (1/2000 or 1/200 serial dilutions). Besides, the level of IgG1 in the mice sera immunized with all formulations was notably (*P*<0.0001) more than that of the HEPES buffer group (1/2000 and 1/200 serial dilutions). The greatest level of IgG was observed in the group of mice immunized by Lip SLA+imiquimod+DOTAP (1/20000 serial dilutions) that was meaningfully (*P*<0.05) higher than those of the other groups (Figure 5C).

***ELISpot results***

**Figure 6:**
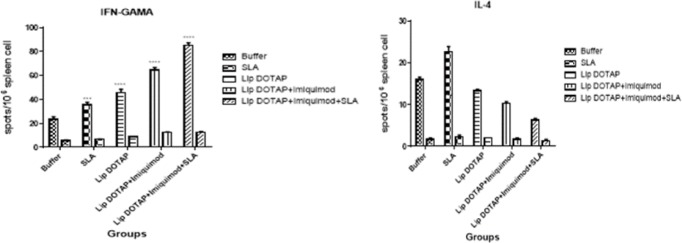
Cytokine level was evaluated through measuring IFN-γ and IL-4 production in immunized mice at week 2 after the last booster injection. Mononuclear splenocytes were cultured in the presence of Soluble *Leishmania* Antigen (10 μg/ml), and the IFN-γ release and IL-4 release from splenocytes induced by different liposomal formulations were determined using ELISpot assay. The data are represented as mean±SEM (n=3)

To evaluate the effectiveness of compositions in the induction of the immune cellular reaction, IL-4 and IFN-γ generation triggered through various liposomal constructs got calculated using the ELISpot experiment. The findings of ELISpot experiments implied that splenocytes removed from the group of mice immunized by SLA, Lip DOTAP, imiquimod+Lip, and Lip+imiquimod+SLA secreted meaningfully (*P*<0.0001) greater IFN-γ amounts (Figure 6), compared to the mice immunized with the HEPES buffer. In contrast, IL-4 production was identifiable in spleen accompanying the stimulation of antigen for all mice groups, but the amounts of IL-4 (Figure 6) did not change significantly in any formulation in comparison with the group of mice that received the HEPES buffer. 

***The results of flow cytometry***

**Figure 7:**
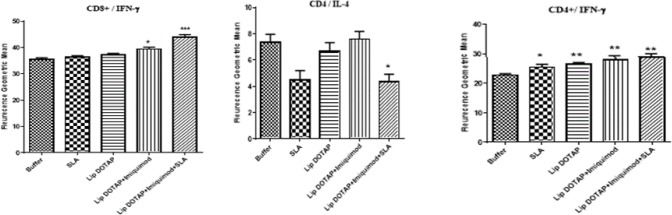
After the last booster, splenocytes were isolated and restimulated, and then stained for surface CD8, CD4, and intracellular IFN-γ and IL-4. Splenocytes were gated by side vs forward scatter light followed by staining with CD8Pe-cy5 and CD4Pe-cy5. Plots show log fluorescence intensity for IFN-γ- FITC and IL-4-PE. The data are represented as mean±SEM, (n= 3). *** (*P<*0.001), ** (*P<*0.01), and * (*P<*0.05) denote significant differences from buffer and all other formulations

In order to determine the antigen reactions specific to the T cell, following the final booster, the separation of splenocytes occurred in various groups of mice. For the surface markers of CD8 and CD4, extra-cellular staining was used. For IL-4 and IFN-γ cytokines, the staining of intracellular cytokines was used in addition to flow cytometric analysis. CD4 and CD8 markers show, respectively, the IL-4 and IFN-γ frequency generating cells in Th1 and Th2 populations. Figure 7 shows that formulations triggered a (*P*<0.01) greater level of IFN-γ generation in CD4^+^ lymphocytes representing the greater quantity of cells generating IFN-γ in the CD4^+^ group compared to the buffer group. The frequency of IFN-γ/CD8^+^ cells in the group of mice immunized by Lip+imiquimod and Lip+imiquimod+SLA was by far (*P*<0.05, *P*<0.001, respectively) more than others. Moreover, flowcytometry revealed generation of IL-4 in the cells of CD4, implying humoral immunity that was T cell-centered. Lip+imiquimod+SLA formulation was (*P*<0.05) meaningfully lower than other groups. 

## Discussion

The development of the *Leishmania *vaccine is a difficult task, mainly as it is impeded by the insufficient familiarity with the pathogenesis of the parasite as well as the immune reaction complexity required for protection purposes. The presence of a proper delivery system to develop an efficient immune reaction against leishmaniasis is the major step in creating an efficient vaccine against it. The current strategies of prophylactic vaccines against intracellular pathogens, such as *Leishmania,* consider enhancing the host’s innate immunity and take into account the pathogen and the adaptive response via the vaccine.

The creation of protective immunity by vaccinations depends on the capacity of the vaccine in extracting the proper immune response capable of monitoring or removing the pathogen. Resisting leishmaniasis is concerned with a Th1 prevailing reaction as well as interleukin-2 (IL-2) and IFN- γ generation by the particular T lymphocyte+CD4 population of the antigen, through mediating protective cellular immune responses (34, 35). Moreover, the activation of the T cell CD8^+^ population exerts a strong effect on preventing getting infected with *L. major* as well as conducting efficient vaccination to fight test murine leishmaniasis (36). Th2 cells generate interleukin-4 (IL-4), interleukin-5 (IL -5), as well as interleukin -10 (IL -10), thereby intensifying the reactions of humoral immunity as well as the uncontrolled progress of susceptibility, metastasis, lesions, and disease exacerbation (37).

Liposomal loads having protein antigens and immune-stimulatory molecules that imitate pathogens in the mode of reductionism could be valuable compositions in producing protective immunity of T cells. Agonists of TLR containing liposomes allow for the simultaneous targeting of the pattern recognition receptor (PRR) pathways and antigen presentation to efficiently develop (effector) T cells (38, 39). 

Accordingly, through the present study, an extremely immuno-stimulating adjuvant composition (imiquimod and cationic liposomes) was provided and utilized in order to develop the soluble antigens of *Leishmania* in the form of the 1^st^ generation of vaccines. The protection level and immunity reactions were investigated in the model of the mice infected with leishmaniasis. To examine the protection rate, several parameters, including the sera’s antibody level, the footpad swelling kinetics, and the burden of parasites in the spleen or footpad of the infected mice were assessed (34). 

The findings implied that the footpad’s swelling size in the group of mice that were immunized by SLA+imiquimod+Lip on day 42 after the challenge was by far (*P*<0.05) lower than those of the rest of formulations (Figure 2). In designing a vaccine against leishmaniasis, the measuring of the foot and splenic burden of parasites is influential as well in measuring the vaccine efficiency. The results of the foot and splenic burden of parasites verified that the mice immunized with Lip+imiquimod+SLA demonstrated a lower burden of parasites than the buffer mice, and no meaningful difference (*P*>0.05) was observed in the parasite numbers of vaccinated groups and the controls (Figures 3A and 3B).

The assessment of IgG2a and IgG1 antigen isotypes was utilized, respectively, as the indicator of Th1 and Th2 immunity responses. In the present study, the IgG2a antigen level in the mice’s sera that were immunized by empty Liposome and SLA+imiquimod+Lip showed to be more than (*P*<0.001) those of other groups before the challenge (Figure 4B). Surprisingly, no meaningful difference was observed in the IgG1 rate between the control and all immunized mice before the challenge (Figure 4A). Although a meaningful difference (*P *>0.0001) existed in the IgG1 levels of all immunized mice and the control group (Figure 5A) after the challenge; quite interestingly, the results showed that only the Lip+imiquimod+SLA formulation improved the IgG2a generation when the challenge was over, being essential in counteracting intracellular pathogens (Figure 5B).

The IFN-γ level, the cytokine implying the Th1 reaction, in evaluating the cytokine demonstrated that all formulations produced a greater level of IFN-γ (*P*<0.0001) compared to the mice that were immunized by the buffer (Figure 6A). In addition, the least amount of IL-4 was recognized inside splenocytes from the mice that were immunized by SLA+imiquimod+Lip, but no meaningful difference was observed in IL-4 levels in different vaccinated groups and the control group (Figure 6B). CD4 and CD8 indicators imply the recurrence of IL-4 and IFN-γ generating cells in Th2 and Th1 groups. The findings demonstrated that Lip+imiquimod and Lip+imiquimod+SLA formulations caused a meaningfully higher IFN-γ level in the lymphocytes of CD8^+^, having been indicative of the larger population of the cells generating IFN-γ in the CD8^+^ group than the rest of the groups (Figure 7A). The recurrence of IFN-γ/CD4^+^ cells of all mouse groups immunized by different compositions was by far higher compared with that of the buffer group (Figure 7B), yet the results of flowcytometry demonstrated that the lowest production rate of IL-4 in the cells of CD4  was induced significantly (*P*<0.05) in the Lip+imiquimod+SLA group in contrast to other groups (Figure 7C).

Lip+imiquimod+SLA could produce a Th1 immunity reaction strategy to secure the mice in the face of leishmaniasis and lead to long-run security against cell infection. The size of the swelling of the footpad, cytokines, as well as the burden of foot and splenic parasites in immunization assays with only SLA did not protect the BALB/c mice. Previous studies revealed that SLA could potentially draw Th2 responses (40). The mixed immune responses by Th2/Th1 following the SLA immunization were similar to those of the past research (41, 42).

A strong immune response was detected after using the liposomal SLA with imiquimod. Although the impact of the boosting imiquimod was greater than that of the cationic liposomes, the reaction was triggered concurrently with the delivery of liposomal Ag. The final difference was detected after the challenge of infection, it seems that the adjuvant combination prevented the multiplication of *L. major* in macrophages when the imiquimod is used to make different formulations of the vaccine. The combination of delivery systems and adjuvants is a new perspective into designing efficient vaccines (35, 43). The results showed that imiquimod-induced Th1-based responses. It was also revealed that the leishmanial Ag vaccination and imiquimod-induced immune protective reactions to the infection challenge of *L. major* in the mice (BALB/c). Thus, the effects of imiquimod might increase in the presence of the vaccine transfer system, including liposomes, generally for the protein-based vaccination; however, purified Ag immunogenicity was strengthened via the cationic liposomal delivery.

Vaccine delivery systems, like liposomes, enhance the related antigen uptake into APCs. The bilayer composition has been found to have a considerable effect on the liposome uptake and interaction via APCs as well as the kind of triggered immune reaction (44-46). Liposome functions as an adjuvant and triggers immune reactions to different antigens. Cationic liposomes protect easily-altered antigens against lysosomal degeneration, so they enjoy the benefits of electrostatic contacts with cells’ negative charges, thereby turning them into a typical location for cells producing antigens (APCs), being critical for the stimulation of immunity (47, 48).

Cationic liposomes, in contrast to neutral and negative liposomes, represent a prevalent depot impact on the site of injection, thereby contributing to delivering the antigens trapped in APCs more forcefully. Triggering the protecting type of Th1 of the immunology reactions (49, 15) is of high significance. Cationic liposomes contain the rgp63 antigen that is absorbed efficiently through BMDCs (i.e., bone-marrow derived dendritic cells) and then carried to different intra-cellular parts. DCs, activated with liposomes containing rgp63, caused effective antigen presentation to the specific cells of CD4^+^ and CD8^+^ T (50).

DOTAP-bearing cationic liposomes show the capacity of this formulation acting as an appropriate antigen delivery method to stimulate T-cell reactions. In selecting efficient immunomodulators, imiquimod is of high importance due to its protective effect on the experimental models of leishmaniasis. Safety and immunogenicity of human’s imiquimod may stimulate APCs via co-stimulatory molecule maturation and extract cytokines, including IFN-γ, IL-4, and IL-12, which are important in terms of activation and maturation of T and B cells. Imiquimod is activated through the immunomodulation impact exerted on various cells that are effective in the immune functions, thereby inducing secretion of cytokines, such as interferon gamma (IFN-γ), the alpha factor of tumor necrosis (TNF-alpha), IL-8 (7–9), IL-6, and interleukin (IL)-1beta. Monocytes and macrophages are the major imiquimod’s target cells (51-53). Imiquimod as well as a similar component, S-28463, triggered the macrophages’ leishmanicidal performance efficiently. Besides, signal transmission concerned with the induction of synthesizing macrophages’ nitric oxide was triggered by imiquimod (54).

The innate immune reaction might be stimulated by different molecular patterns associated with pathogens through the toll-like (TLR) type of receptors, having been effective in regulating the immune reaction produced (55). From 11 TLRs (mammalian), TLRs 9, 8, 7, and 3 are present inside the endosomes of cells that identify the DNA pathogens and intracellular RNA’s nucleic acids (56, 57). By detecting pathogens, or their constituents using the TLRs, the premature antigen-producing cells, including DCs (dendritic cells), get matured and move to the node of the draining lymph. The developed DCs demonstrate the antigens obtained; they also activate ASTC (antigen-specific T cells), thereby triggering the creation of the immunity specific to antigens accompanied by the immunologic memory. The immunity produced is to be influenced, leading to the stimulation of TLR(s) and the particular DC subset (55). Utilizing the Toll-like receptor (TLR) agonists as the adjuvants of vaccines indicates the influential approach to developing vaccines with enhanced protective immunity. Considering the adjuvant features of the topical imiquimod in treating infectious ailments, it was examined in the mice (BALB/c) as a vaccine adjuvant in a CL experimental model. It was revealed that the topical imiquimod’s subcutaneous use on skin before it was immunized by the crude antigen of *Leishmania* enhanced the prevention of the infection challenge compared with the immunization of the crude antigen, being correlated with an improved Th1 reaction contrary to the vaccine antigen (58). It was also shown that the imiquimod’s topical utilization at the subcutaneous injection of CS* (Plasmodium falciparum* circumsporozoite) peptide triggered potent responses of Th1 and large amounts of antibody concentrations that led to security against the infection challenge (59). Further attention must be paid to the use of topical imiquimod. which is used in the form of an adjuvant of the vaccine for every subcutaneously delivered antigen of pathogens, especially when the Th1 reaction must be triggered for protective immunity.

## Conclusion

In general, the results demonstrated that Cls (cationic liposomes) were adjuvant factors effective in protecting against the challenge of *L. major* in mice (BALB/c), yet more powerful CMI (cell mediated immunity) reactions became activated where imiquimod was contained in liposomes. Hence, immune modulation by cationic liposomes could be effective in improving immunization against *L. major*, suggesting that future vaccine treatments for *Leishmania*, the effects of lipid compositions, and immunostimulatory adjuvants must be considered in terms of the kind of immune reactions that must be triggered.
